# Streptomyces phaeochromogenes BV-204, K-1115A Anthraquinone-Producing Strain: A New Protein Biosynthesis Inhibitor

**DOI:** 10.32607/actanaturae.27315

**Published:** 2024

**Authors:** A. R. Belik, Yu. V. Zakalyukina, V. A. Alferova, Y. A. Buyuklyan, I. A. Osterman, M. V. Biryukov

**Affiliations:** Sirius University of Science and Technology, Sochi, 354340 Russian Federation; Lomonosov Moscow State University, Moscow, 119234 Russian Federation; Shemyakin–Ovchinnikov Institute of Bioorganic Chemistry, Moscow, 117997 Russian Federation; Skolkovo Institute of Science and Technology, Moscow, 121205 Russian Federation

**Keywords:** actinomycetes, K-1115A, antibiotics, reporter system pDualrep2, inhibition of protein biosynthesis, in vitro translation, citizen science

## Abstract

In the search for new antibiotics, it is a common occurrence that already known molecules are “rediscovered” while new promising ones remain unnoticed. A possible solution to this problem may be the so-called “target-oriented” search, using special reporter microorganisms that combine increased antibiotic sensitivity with the ability to identify a molecule’s damaging effect. The use of such test organisms makes it possible to discover new promising properties even in known metabolites. In this study, we used a high-throughput screening method based on the pDualrep2 dual reporter system, which combines high sensitivity through the use of modified strains of test organisms and makes it possible to easily and accurately identify the interaction mechanisms of a substance and a bacterial cell at the initial stages of screening. This reporter system is unknown in Russia and is significantly superior to its global analogues. In the system, translation inhibition induces the expression of the fluorescent protein Katushka2s, while DNA damage is induced by TurboRFP. Using pDualrep2, we have isolated and described BV-204, an *S. phaeochromogenes* strain producing K-1115A, the biologically active substance that we have previously described. In our study, K-1115A for the first time has demonstrated antibiotic activity and an ability to inhibit bacterial translation, which was confirmed *in vitro *in a cell-free translation system for FLuc mRNA. K-1115A’s antibacterial activity was tested and confirmed for *S. aureus *(MRSA) and *B. subtilis*, its cytotoxicity measured against that for the HEK293 cell line. Its therapeutic index amounted to 2 and 8, respectively. The obtained results open up prospects for further study of K-1115A; so, this can be regarded as the basis for the production of semi-synthetic derivatives with improved therapeutic properties to be manufactured in dosage forms.

## INTRODUCTION

Pathogens becoming more resistant to antibiotics is one of the most pressing problems of modern medicine, because the potential of the molecules already found and long ago introduced into medical practice is now almost exhausted, and the rate of discovery of new ones has significantly slowed compared to what it was during the “Golden Age” of antibiotics in the middle of the twentieth century. Most antibiotics discovered during large-scale screening [1, 2, 3] turn out to be “rediscoveries” of previously discovered molecules, but the new tools for investigating action mechanisms have enabled us to look at these substances from a new angle and discover new potential in them [4, 5, 6].

Such new tools are target-based screening and the methods for determining the action mechanism of a molecule at the initial stages of research. These tools allow one to focus a search on the antibacterial agents specific to the most promising targets and may even accelerate molecule identification. Currently, we are successfully utilizing a reporter system in which compounds that inhibit protein or DNA biosynthesis are detected by fluorescent protein reporter gene expression in response to this inhibition. The relevance of this approach lies in that a ribosome is a key element in the functioning of a living cell, and considering the significant differences in the structures of the ribosomes of pro- and eukaryotes, it creates an opportunity to produce a highly specific effect on bacterial ribosomes and significantly increase the chances of developing drugs with good therapeutic properties.

Actinomycetes of the genus *Streptomyces *are the richest source of biologically active substances and produce approximately 50% of the antibacterial substances used in pharmaceutics [7, 8, 9]. Actinomycetes are among the prokaryotes of the largest genomes and have a large coding potential imparting a structural diversity to the secondary metabolites they produce. Tens of thousands of such molecules have been described so far, and even “rediscovered” ones often show new, unique properties.

Previously, thanks to the mentioned reporter system, we were able to establish the action mechanism of tetracenomycin X [10], a molecule whose antibacterial properties were first described in the 1960s. Tetracenomycin X has a structure similar to that of doxorubicin, so it was believed that its effect was also based on intercalation into the double-stranded DNA structure. But our study showed that this molecule inhibits protein biosynthesis by interacting with the ribosome in a new, previously unexplored binding center, giving hope that new, promising semi-synthetic derivatives may be developed from it.

In the present study, we detected a strain producing K-1115A, a substance whose antibacterial action mechanism is based on protein biosynthesis inhibition. This was confirmed by a test in a cell-free translation system. The results obtained have allowed us to conclude that the substance acts as an inhibitor of protein biosynthesis.

## EXPERIMENTAL

**Microorganisms sampling, isolation and culturing**

*S. phaeochromogenes *BV-204 was isolated from soil samples collected at the Sirius Federal Territory. The samples were collected in the spring of 2021 in a park area on the Black Sea coast (43°23’53.7″N 39°57’48.2″E). Sampling was carried out following the method described in [11, 12]. The top layer of the soil (0–5 cm) was removed with a sterile spatula and placed in a sterile specimen collection container. Actinobacteria were isolated by surface seeding on agarized nutrient media from serial dilutions of soil suspensions as per [13]. ISP3 [13] combined with nystatin (250 µg/mL) and nalidixic acid (10 µg/mL) was used as a nutrient medium to inhibit the development of fungi and Gram-negative bacteria, respectively. The culture was incubated for 14 days at 28°C.

Strain BV-204 was selected on the basis of morphological characters, isolated in pure culture from primary inoculation on the Gauze 1 mineral agar for micromorphological studies [14]. For *in vitro *maintenance, the strain was cultured on the ISP3 medium; for long-term storage, it was grown in a liquid ISP3 medium for 14 days with constant stirring (200 rpm at 28°C), and then the resulting suspension was mixed with an equal volume of a 50% glycerol solution and frozen in liquid nitrogen; the samples were stored at -80°C.

**Polyphase strain identification**

The culture attributes of the strain (presence and color of aerial mycelium, release of soluble pigments) were evaluated on dense media recommended by the International Streptomyces Project (ISP) after 14 days of cultivation at 28°C [15]. The morphological characteristics (presence and shape of reproductive spore chains, character of the spore surface) were evaluated using a Zeiss Axiolab A1 light Zeiss (Carl Zeiss Microscopy GmbH, Germany) and a scanning electron microscope JSM-6380LA (JEOL Ltd., Japan) after 14 days of growth at 28°C in the ISP3 medium. The samples for electron microscopy were prepared as described in [16]. The utilization of carbon sources (mono- and polysaccharides, alcohols) was evaluated on a ISP9 mineral agar with the addition of bromocresol purple at 28°C during 14 days [15]; the ability to degrade starch, cellulose, and casein by the size of polymer hydrolysis zones, as per [17, 18]. The sensitivity to different antibiotics was determined using antibiotic-impregnated paper disks (HiMedia Laboratories Pvt. Ltd., India).

**Whole-genome sequencing, phylogenetic analysis and BGC analysis**

DNA from the producer strain was isolated as per [19]. The genome of strain BV-204 was sequenced *de novo *on an Illumina HiSeq 4000 platform (Illumina, USA) and assembled using SPAdes v3.13.0 [20]. It was annotated using the RASTtk pipeline based on the PATRIC web service [21]. The genome’s integrity and quality, as well as average nucleotide identity (ANI), were assessed using the MiGA web service (http://microbial-genomes.org). Its phylogenetic affiliation was investigated using full-genome sequencing on the Type (Strain) Genome Server (TYGS) (https://tygs.dsmz.de/). The genome was automatically compared to all the genomes represented in the TYGS database using the MASH algorithm [22]. Its phylogenetic tree was obtained using FastME 2.1.6.1 based on GBDP distances calculated from the genome’s nucleotide sequences. The branch lengths were scaled by applying the GBDP d5 distance formula [23]. The BGCs of its bioactive compounds were identified using the bacterial version of the antiSMASH 6.1.0 browser (https://antismash.secondarymetabolites. org). Homologous regions in each genome were identified using NCBI Blastn (https://blast.ncbi. nlm.nih.gov).

**Antimicrobial action screening**

The primary antibacterial activity was determined on* E. coli *BW25113, whose codons 330–352 of the lptD gene were deleted, so it is hereinafter referred to as* E. coli *SS_lptd. This mutation leads to disruption of the normal lipopolysaccharide envelope synthesis of Gram-negative bacteria, making it more permeable to low molecular weight compounds [11]. The strain contains the pDualrep2 plasmid [10]. In the presence of DNA replication or protein biosynthesis inhibitors, the strain expresses the fluorescent protein TurboRFP or Katushka2S (Supplementary, *Fig. S7*). Screening was performed by the agar diffusion method described previously [16]. Along with other strains, strain BV-204 was grown on ISP3 and tested on days 3, 6, and 9; for this purpose, a 5-mm-diameter agar block was cut from a lawn area with distinct growth and placed on cups containing an agarized LB medium pre-cultured with the test organisms. The fluorescent signal was detected the next day after culturing using a ChemiDoc MP (Bio-Rad) in SU 3 and SU 5 channels. To study the strain’s action spectrum, BV-204 was tested on other test organisms, such as *S. aureus* ATCC 29213, *S. aureus *ATCC 25923, *S. aureus *SS01,* S. aureus *(MRSA) INA00761, *B. subtilis *ATCC 6633,* C. albicans *CBS 8836, and *M. smegmatis *Ac-1171. The antibacterial activity was evaluated by applying the agar diffusion technique described above. To form a bacterial lawn, an Agarized LB medium was utilized. As for yeast, it was glucose-peptone-yeast agar [24], incubated at 37°C for 24 h to evaluate the size of growth suppression zones.

**Separation and identification of active components**

Initial screening established BV-204 ability to exhibit antagonistic activity when grown on the ISP3 medium. To obtain the CF containing the active ingredient, the strain was cultured on a ISP3 liquid nutrient medium (7 days, 28°C in a New Brunswick Innova shaker (Eppendorf) at 200 rpm). The CF was separated from the biomass by centrifugation at 4, 000 g, concentrated and purified by SPE. For this purpose, CF was applied to a Poly-Prep Econo-Pac chromatographic column (Bio-Rad) containing 1 ml of LPS- 500H sorbent (Tekhnosorbent, Russia), then eluted with a stepwise gradient of water-acetonitrile (v/v) for fractional collection of the eluate. The antagonistic activity of the collected fractions was investigated, and their active fractions were purified using HPLC.

HPLC analysis and fractionation were performed in a Vanquish Flex system with a diode array detector (Thermo Fisher Scientific, USA) equipped with a 5 μm C18(2) 100 Å, 250 × 4.6 mm Luna column (Phenomenex), flow rate 1 mL/min, injection volume 20 μL. A 0.1% aqueous THF solution was used as eluent A and acetonitrile with 0.1% THF as eluent B. Elution was performed by increasing the eluent B concentration from 25 to 95% for 10 min and then maintaining it at 95% for 2 min. Fractions of 1 mL were collected to analyze their antibacterial activity (*Fig. S8*).

The active fractions were analyzed using a UltiMate 3000 chromatograph (Thermo Fisher Scientific, USA) equipped with an Acclaim RSLC 120 C18 2.2 μm 2.1 × 100 mm column (Thermo Fisher Scientific) and an amaXis II 4G ETD qTof-mass spectrometer (Bruker Daltonics). Measurements were performed in the 100–1,500 m/z spectrum recording mode to register the three most intense ions, dissociation type CID 10–40 eV, in nitrogen. The mass spectra were analyzed using OpenChrom Lablicate Edition (1.4.0.202201211106), TOPPView v.2.6.0 [25]. Chemical structures were identified using the GNPS [26], NPAtlas [27, 28], and the Dictionary of Natural Products 31.1 databases.

The active HPLC fraction (1 mL) was concentrated using a CentriVap vacuum bioconcentrator (Labconco) and dissolved in 500 μL of a 10% aqueous DMSO solution; the resulting solution was referred to as the “antibiotic working solution.”

**Translation inhibition *in vitro***

Translation suppression was studied in a cell-free system using a commercial *E. coli *T7 S30 Extract System for Circular DNA kit (Promega) as per the manufacturer’s instructions. The Antibiotic solution (0.5 μL) was added to the reaction mixture (4 μL) followed by 0.5 μL of 200 ng/μL FLuc mRNA and incubated for 1 h at 37°C.

The luciferase activity was measured by chemiluminescence intensity at a wavelength of 580(80) nm using the Luciferase Assay Reagent kit (Promega) in a ClarioStar plate reader (BMG Labtech).

**MIC and cytotoxicity**

Overnight cultures of *E. coli *SS_lptd, *S. aureus* INA00761 (MRSA), *S. aureus *SS01, and *B. subtilis* ATCC 6633 were diluted with a fresh LB medium to OD_600_ = 0.6, and then the resulting inoculum was diluted 1,000-fold to obtain a working suspension. Some 100 µl of the suspension was added to the wells of a sterile 96-well plate, except for the first and last rows. The first row was filled with 180 μl of the suspension each, and the last row was filled with 100 μl of a sterile nutrient medium to be used as a negative control. Then, 20 μl of the antibiotic solution was added to the first row of the plate and a series of twofold dilutions were obtained by sequentially transferring 100 μl from the well of one row to the well of the next row. The penultimate row, where no antibiotic was delivered, was used as a positive control. The plate was then incubated under stirring (200 rpm, 37°C). Cell growth was recorded in 24 h at a wavelength of 590 nm in the ClarioStar tablet reader. The substance concentration completely suppressing bacterial growth was considered as MIC.

To determine the cytotoxicity, the cell lines were prepared as per [29]. The HEK293 cells were cultured on the DMEM nutrient medium containing 10% FBS, 4 mM of L-glutamine, and 4.5 g/L of glucose. A row of prepared microcentrifuge tubes was filled with 100 µl of the nutrient medium each, then 80 µl of the medium and 20 µl of the antibiotic solution were added to the first tube, followed by two-fold serial dilutions, transferring 100 μl each from the first tube to the second tube and then throughout the row. Double dilutions of doxorubicin ranging from 75.9 to 0.16 μM were used as negative controls; a number of wells with cells without antibiotics were left as positive controls. The contents of the tubes were transferred to the corresponding wells of a pre-arranged plate with cells and incubated for 3 days in a CO_2_ incubator at 37°C. After the incubation, 20 μL of a resazurin solution (0.15 mg/mL) was added to the wells containing a nutrient medium, stirred by rocking to distribute the dye evenly and incubated in a CO_2_ incubator at 37°C for 3 h. Fluorescence intensity was then measured in the ClarioStar plate reader (Ex = 545 nm, Em = 600 nm).

## RESULTS

**Genetic and phylogenetic analyses**

**Fig. 1:**
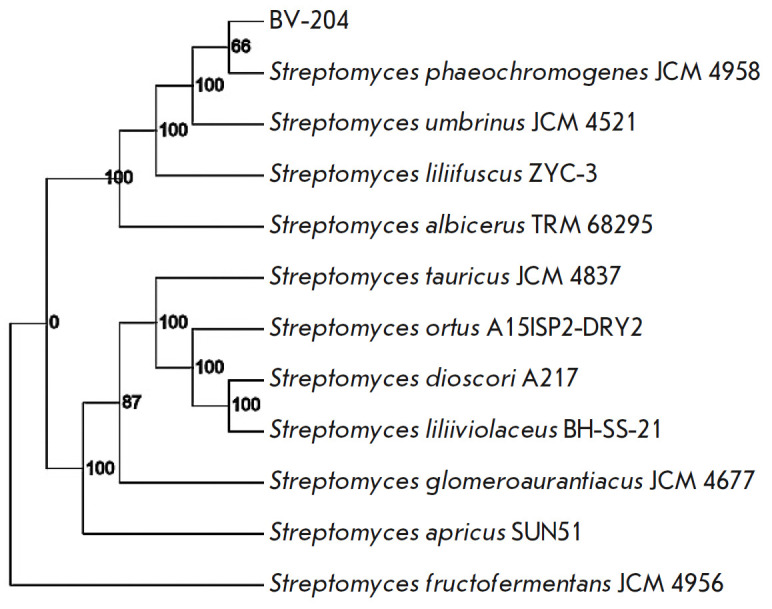
Phylogenetic tree based on the complete genome of *S. phaeochromogenes *BV-204. The bootstrap analysis values are above 60%

The results of full-genome sequencing and subsequent assembly demonstrated the genome size of BV-204 cells to be typical for representatives of the genus *Streptomyces *[30] and comprised 11,380,121 bp at the G+C content of 70.2%. Phylogenetic analysis showed that BV-204 clustered most closely with* S. phaeochromogenes *JCM 4958 (formerly *S. ederensis* JCM 4958) and together with it, as well as with* S. umbrin*us JCM 4521, *S. liliifuscus *ZYC-3, and* S. albicerus *TRM 68295, formed a monophyletic group with a maximum branching support value of 100% (*Fig. 1*).

**Phenotypic and morphological properties of BV-204**

The strain is a Gram-positive aerobic bacterium with immobile cells growing actively on the ISP2 and ISP3 and moderately on the ISP5 and ISP6 nutrient media. The substrate mycelium coloration varies from dark-brown to beige, the aerial mycelium is pale with pink shades, and no aerial mycelium is formed on the ISP6 medium. In addition, the strain growing on the ISP3 medium produces a dark brown soluble pigment (Supplementary, *Table S1*).

BV-204 cells have the same spectrum of carbohydrate utilization as previously described *S. phaeochromogenes* JCM 4958(T) and *S. umbrinus *JCM 4521: they show no differences in their ability to utilize mono-, disaccharides, and alcohols. We also found that BV-204 was able to hydrolyze carboxymethylcellulose; i.e., this strain possesses cellulase activity not previously encountered in other representatives of this taxon (Supplementary, *Table S2*).

**Fig. 2:**
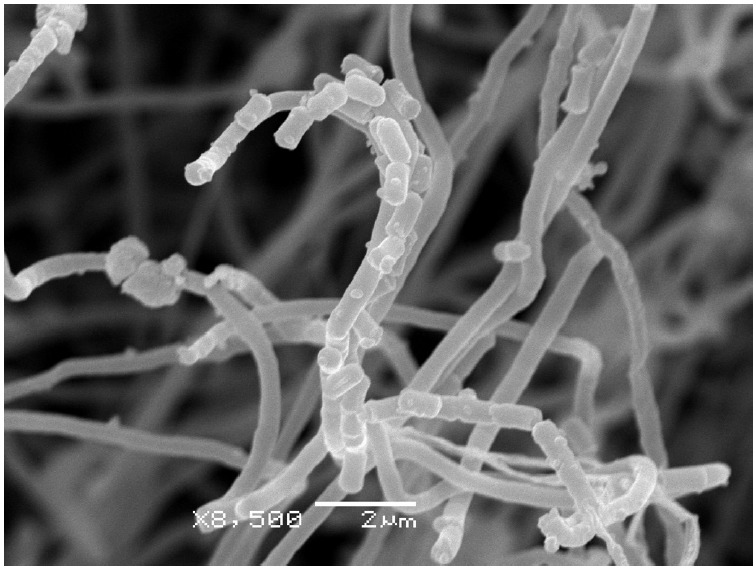
Electron micrography of *S. phaeochromogenes* BV-204 on the 14^th^ day of incubation on the ISP3 medium at 28°C. The dimensional cutoff is 2 μm

BV-204 forms straight, long chains of spores with a smooth surface, typical for strains of its kind [14] (*Fig. 2*).

Therefore, the results of the phylogenetic analysis based on the polyphasic taxonomic approach and comparison of phenotypic traits enabled us to assign BV-204 to the species *S. phaeochromogenes.*

**Antibacterial activity**

**Fig. 3:**
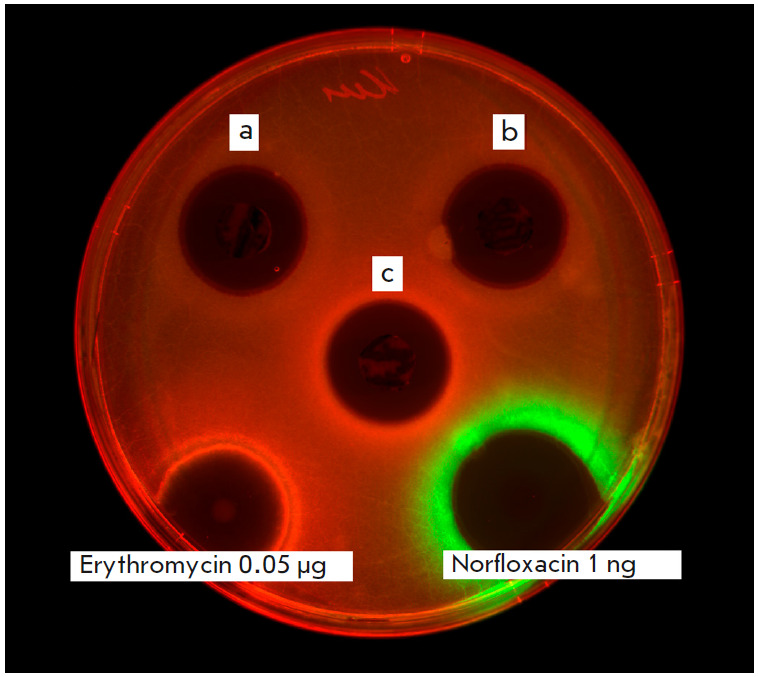
Activity of the agar blocks containing* S. phaeochromogenes *BV-204 in the presence of *E. coli* lptd pDualrep2 on (a) the 3^rd^, (b) 6th, (c) and 9th days of growth

Initial screening revealed the strain’s antibacterial activity against *E. coli *SS_lptd pDualrep2. The substance secreted by the producer induced Katushka2S expression that could possibly inhibit protein synthesis (*Fig. 3*). As positive controls, we used 0.05 μg of erythromycin (protein biosynthesis inhibitor) inducing Katushka2S expression, and 1 ng of norfloxacin (DNA gyrase inhibitor) inducing TurboRFP expression. For convenience, the Katushka2S and TurboRFP signals were visualized in respective red and green by the ChemiDoc MP software. In *E. coli *JW5503 ΔtolC pDualrep2 no inhibition or induction of reporter fluorescent proteins was registered.

BV-204 was found to inhibit the growth of Grampositive bacteria *B. subtilis *ATCC 6633, *S. aureus *SS01, and *S. aureus *INA00761 (MRSA); however, it did not inhibit *S. aureus *ATCC 29213, *S. aureus *ATCC 25923,* C. albicans *CBS8836, or *M. smegmatis *Ac-1171. Both the agarized and liquid ISP3 media were deemed optimal for the strain to synthesize active metabolites.

**Active substance identification**

**Fig. 4:**
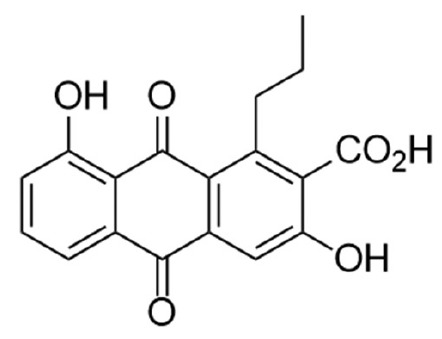
Structure of the identified active component K-1115A (3,8-dihydroxy-9,10-dioxo-1-propylanthracene- 2-carboxylic acid)

Pure active metabolites were obtained by solid-phase extraction from *S. phaeochromogenes *BW-204 CF and detected in fractions containing 30–40% acetonitrile. The fractions were concentrated and subjected to further separation and fractionation by HPLC. The activity was found to be associated with a component eluted at 9.47 min (Supplementary, *Figs. S10 *and* S11*) and with absorption maxima at 276 and 407 nm. CMS analysis of this substance showed that it almost did not get ionized in the positive ion detection mode but yielded an intense [M–N] adduct corresponding to an accurate mass of 326.0805 Da (Supplementary, *Fig. S12*). Considering the characteristic absorption spectrum of the isolated compound in the NPAtlas, Dictionary of Natural Products, and PubChem databases, a candidate with the gross formula C18H14O (accurate mass 326.0790, deviation 4.5 m.d.) and the structural formula shown in *Fig. 4* was found.

As alnumycin, K-1115A is of biosynthetic origin and produced by streptomycetes [31]. The fragmentation spectrum of the adduct with *m/z *325.07 showed a major fragment ion [M–44] with *m/z *281.08, consistent with the carboxyl group present in the molecule.

**Inhibition of protein biosynthesis by K-1115A *in vitro***

**Fig. 5:**
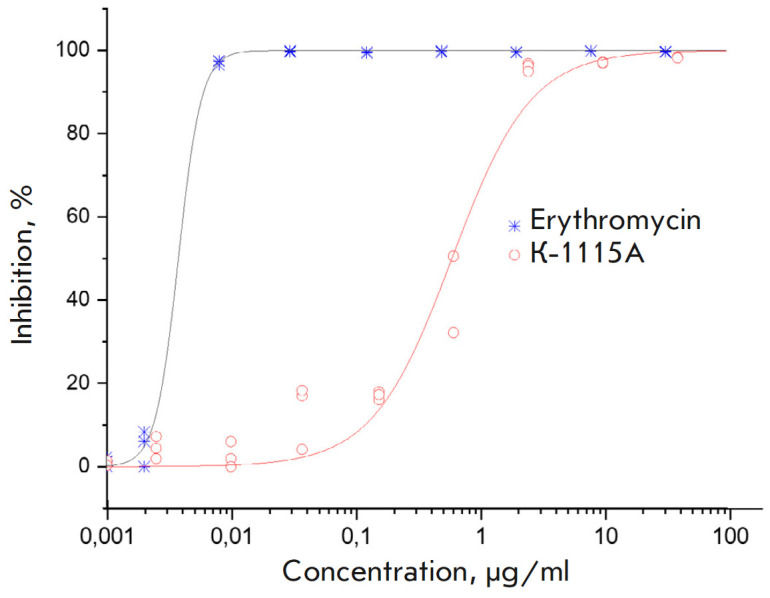
*In vitro *inhibition of luciferase gene translation by K-1115A and erythromycin

The ability of the HPLC-purified fraction of K-1115A to inhibit cell-free translation was investigated with erythromycin used as a reference translation inhibitor. The experiments were carried out in triplicate; the results of determining the dose-effect concentration dependence are presented in *Fig. 5*. The EC_50_ values were 0.004 and 0.606 µg/mL for erythromycin and K-1115A, respectively.

**BGC analysis**

**Fig. 6:**
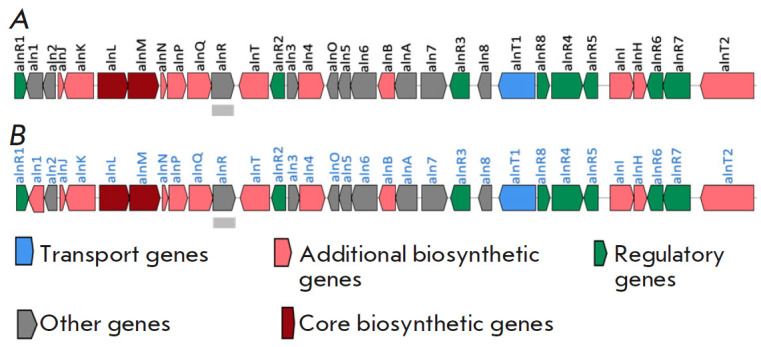
Alnumycin and K-1115A BGC in the genomes of (*A*) *Streptomyces sp*. CM020; (*B*) *S. phaeochromogenes* BV-204

The bioinformatic methods applied enabled us to detect alnumycin BGC in the genome of BV-204 with K-1115A as a bypass product, which was homologous to the gene cluster previously annotated in* Streptomyces *sp. CM020 (*Fig. 6*) [32]. The BGC contains 32 open reading frames that may participate in the biosynthesis of alnumycin and K-1115A. It presumably consists of 22 structural and 10 regulatory and transport genes. The cluster’s total length is 31,030 bp. The antibiotic is synthesized by a type-II polyketide synthase.

## DISCUSSION

The genus *Streptomyces *is the most extensive among actinomycetes, so most of the known and clinically relevant antibiotics, starting from streptomycin, have been isolated from the members of this taxon. Through the efforts of numerous scientific teams, data on tens of thousands of compounds exhibiting antagonistic activity had been published by 1980; most of them, however, were not fully characterized due to the limited methodological base of that time. One such compounds turned out to be K-1115A produced by the BV-204 strain that we detected during a large-scale citizen-science screening using the pDualrep2 target-based reporter system.

The BV-204 producer strain, attributed from a polyphase analysis to the *S. phaeochromogenes *species, demonstrated the ability to inhibit model strains and cause induction of a reporter system, suppressing protein synthesis when grown on an oat medium.

Successive stages of CF solid-phase extraction followed by HPLC fractionation resulted in the isolation of the pure active substance. Subsequent CMSA suggested K-1115A (3,8-dihydroxy-9,10-dioxo-1-propylanthracene- 2-carboxylic acid) as a candidate, as evidenced by the exact mass match and characteristic UV spectrum consistent with previously published data [33]. The bioinformatic analysis revealed that the BV-204 genome had a BGC responsible for the production of alnumycin and other related substances, including K-1115A, which also confirmed the strain’s ability to synthesize K-1115A. Moreover, the antibacterial activity of K-1115A had not been previously reported.

Some products of alnumycin BGC are known to have antibacterial activity [31], but their action mechanism has not been studied yet. Analysis of the reference genome (GenBank RefSeq:GCF_026343615.1) of* S. phaeochromogenes *NBC 00034 also revealed it to have an alnumycin BGC and conjugated compounds. This cluster had been annotated previously based on* Streptomyces *sp*. *CM020 [32], whose phylogenetic attribution data have not been published. However, considering the high homologic and phenotypic similarity of the alnumycin BGCs from *Streptomyces *sp*. *CM020,* S phaeochromogenes *NBC 00034, and *S phaeochromogenes* BV-204, it can be assumed that *Streptomyces *sp*.* CM020 also belongs to the *S. phaeochromogenes *taxon.

According to published data, *S. phaeochromogenes* NBC 00034 produces various isomers of phaeochromacetin (A, B, C, D, E) [34, 35, 36, 37, 38], as well as moenomycin and bambermycin [38], but CMSA did not detect these products in the spectrum of the BV-204 metabolites. It is noteworthy that no alnumycin was detected either. Disruption of the *aln4 *and *aln5 *genes can affect alnumycin biosynthesis [34] and transform it to K-1115A as a major product [32]. Gene alignment demonstrated that *aln4 *in *Streptomyces sp. *CM020 and *S. phaeochromogenes *NBC 00034 were fully identical, whereas the nucleotide sequence of the *aln4* gene of BV-204 differs from them by several substitutions. The genetic sequences of the *aln5 *gene in the analyzed strains differ much more strongly (Supplementary, *Fig. S9*). This is probably the reason why our strain produces only K-1115A.

Analysis of the BV-204 genome revealed the regions responsible for the synthesis of many secondary metabolites, such as siderophore coelichelin, but investigating the strain’s ability to form iron-chelating compounds using the agar diffusion method [39] yielded negative results.

3,8-dihydroxy-9,10-dioxo-1-propylanthracene-2-carboxylic acid was first isolated from the *S. griseorubiginosus* strain (Mer-K1115A). It was described as an anthraquinone-series compound and named K-1115A [33]. The compound’s anti-inflammatory properties have been studied, but no data on its antimicrobial activity have been reported in the literature. However, there have been publications reports on the antagonistic activity against Gram-positive bacteria of a structurally similar molecule (3,8-dihydroxy-1-propylanthraquinone- 2-carboxylic acid) [40]. Discs loaded with 40 μg of the active agent were placed on the *S. aureus* and *S. viridochromogenes *lawns, with inhibition zones of 14 and 12 mm in diameter, respectively, and no activity against yeasts and micromycetes was detected [40]. Here, it should be noted that, probably by mistake, the authors named the molecule they studied “K-1115A” despite it having a different formula. Another closely related compound (3,8-dihydroxy-lmethylanthraquinone- 2-carboxylic acid) inhibited the biofilms formed by methicillin-resistant *S. aureus *at EC_50_ at 200 μg/mL [41]. It appears necessary to mention that this study was referenced in the recent paper erroneously attributing the antibacterial activity to K-1115A. However, the present paper is where for the first time the antibacterial activity of K-1115A has been reliably demonstrated. There is evidence that alnumycin and some other BGC products, such as 6-dihydro-8-propylanthraquinone, are active against* E. coli *ΔtolC [42], but in our experiments K-1115A showed no activity against this pathogen. K-1115A affected a variant of the *E. coli *SS_lptd strain with impaired synthesis of envelope lipopolysaccharides and increased cell membrane permeability. According to published data, anthraquinone compounds mainly affect Gram-positive microorganisms, in particular* Staphylococcus, Bacillus, Streptomyces, *etc., but not Gram-negative bacteria or fungi [40].

Anthraquinone substances generally inhibit the processes involved in DNA biosynthesis, but the products and intermediates of alnumycin BGC, in particular alnumycin itself and 1,6-dihydro-8-propylanthraquinone, do not bind to double-stranded DNA and do not inhibit DNA gyrase [43]. Examination of K-1115A using the pDualrep2 reporter system also found no inhibitory effect on DNA synthesis but revealed its ability to inhibit protein biosynthesis.

We studied the effect K-1115A had on translation in a cell-free system *in vitro *and demonstrated that this compound acts as an inhibitor of protein biosynthesis. For K-1115A, EC_50_ was found to be 97% of its MIC, while that of erythromycin in this study was 0.8%. In other words, the concentration of erythromycin must be 125-fold higher to inhibit cell growth than to inhibit translation, whereas in the case of K-1115A these concentrations are almost identical. This property somehow distinguishes K-1115A from common translation inhibitors, but it does not make it unique. For example, chloramphenicol, a well-known translation inhibitor having additional action mechanisms besides binding to the ribosome, has a similar effect [44]. This may suggest that inhibition of bacterial translation is not the only mechanism of antimicrobial action that K-1115A possesses. For instance, some enzyme systems in a microbial cell may modify K-1115A in such a way that the resulting molecule has a significantly greater inhibitory effect that cannot be observed *in vitro*.

It may also be assumed that disrupting bacterial cell membrane synthesis is not an alternative action mechanism, since in our case the substance affects a strain with already disrupted LPS synthesis, but not a strain in which this structure is preserved. Another assumption is that the additional target is not related to the interaction with DNA, even though this is typical for anthraquinone derivatives; otherwise, we would have observed an additional induction of TurboRFP in the experiment, indicative of an SOS response.

It should be noted that K-1115A’s specificity against Gram-positive bacteria and a recombinant *E. coli* SS_lptd strain with a deletion in the lptD gene indicates that its activity against bacterial cells is largely determined by cellular barriers permeability.

For a compound to be considered as a perspective agent of antimicrobial therapy, it is its therapeutic index (TI) indicating the specificity of the effect a compound has on pathogenic and animal cells that plays the major role. For that purpose, we examined the ability of K-1115A to inhibit the HEK293 cell line (EC_50_ of 5 μg/mL) and several model pathogens (*Table 1*) to calculate its TI as the ratio of HEK293 EC_50_ and microbial-strain MICs.

**Table 1:** K-1115A capability to inhibit model pathogenic strains

Tested strain	MIC, µg/mL	TI
E. coli SS_lptd	0.625	8
S. aureus (MRSA) INA00761	2.5	2
B. subtilis ATCC 6633	1.25	4
S. aureus SS01	0.625	8
S. aureus 29213	No*	–
S. aureus ATCC 25923	No	–
M. smegmatis Ac-1171	No	–
C. albicans CBS 8836	No	–

^*^No inhibition in the range investigated.

For the inhibited strains, TI varied from 2 to 8, which gives hope that semi-synthetic derivatives with good clinical prospects can be developed based on K-1115A.

We also obtained evidence that that the products and intermediates of alnumycin BGC had antitumor activity through paraptosis [45], autophagy [45], prevention of aberrant cellular metabolism [46], increased radiosensitivity of cancer cells [47], apoptosis, etc. [48]. K-1115A was shown to have anti-inflammatory properties; it inhibited direct binding of the AP-1 transcription factor to the AP-1 oligonucleotide and collagenase production in IL-1a-stimulated rat synovial cells *in vitro*. It was also found that K-1115A attenuates the AR-1-mediated inflammatory response by reducing the activity of ornithine decarboxylase in mice induced by phorbolmyristate acetate [31]. Patterson et al. have used substance K-1115A to produce physiologically active conjugates [49].

## CONCLUSION

The use of modern mechanism-oriented approaches in classical screening makes it possible to identify not only new biologically active substances, but also to discover new promising properties of previously discovered, but poorly studied, molecules, giving them a chance to become potential drugs. Despite the fact that K-1115A was discovered more than 25 years ago and that data on the antibacterial activity of its homologs have long been available, the activity of this substance and its action mechanism have not yet been studied. In this study, using the pDualrep2 dual reporter system, we were able to detect a K-1115A producer, establish that this compound inhibits protein synthesis, and confirm this effect by inhibiting translation* in vitro *using FLuc mRNA.

K-1115A, an anthraquinone derivative produced by *S. phaeochromogenes *BV-204, has been shown to affect the *E. coli *SS_lptd strain with an attenuated cell membrane and subsequently a number of clinically relevant *S. aureus *isolates, including MRSA and* B. subtilis*. No activity against *S. aureus *ATCC 29213,* S. aureus *ATCC 25923, *C. albicans *CBS 8836,* M. smegmatis *Ac-1171 gives hope K-1115A may turn out to be effective (in terms of specificity) against bacterial targets. K-1115A has also demonstrated a novel property for alnumycin BGC products; namely, the ability to inhibit protein biosynthesis.

The substance’s TI is relatively small (2–8 in different strains), but K-1115A may become the basis for the development of semi-synthetic derivatives for antimicrobial therapy. Earlier studies found K-1115A and its semi-synthetic derivatives to have an anti-inflammatory effect, which, together with its antimicrobial properties, allows us to consider them as promising compounds for the development of preparations for complex antimicrobial and anti-inflammatory therapy (e.g., for wound treatment).

High-throughput screening using the pDualrep2 system has significantly improved efficiency when searching for new protein-synthesis inhibitors even among known compounds, opening up new properties and perspectives for entire classes of molecules.

## Abbreviations

BGC: biosynthetic gene cluster
CF: culture fluid
SPE: solid phase extraction
MIC: minimum inhibitory concentration
CMSA: chromatography-mass spectrometric analysis
HPLC: high-efficiency liquid chromatography
LPS: lipopolysaccharide
FLuc mRNA: messenger RNA encoding firefly luciferase

## References

[1] Abdel-Razek A.S., El-Naggar M.E., Allam A., Morsy O.M., Othman S.I. (2020). Processes..

[2] Newman D.J., Cragg G.M. (2020). J. Nat. Prod..

[3] Dai J., Han R., Xu Y., Li N., Wang J., Dan W. (2020). Bioorganic Chem..

[4] Wright G.D. (2012). Chem. Biol..

[5] Zhu Y., Huang W.E., Yang Q. (2022). Infect. Drug Resist..

[6] Alferova V.A., Maviza T.P., Biryukov M.V., Zakalyukina Y.V., Lukianov D.A., Skvortsov D.A., Vasilyeva L.A., Tashlitsky V.N., Polshakov V.I., Sergiev P.V. (2022). Biochimie..

[7] Mahajan G.B. (2012). Front. Biosci..

[8] Sousa J.A.D.J., Olivares F.L. (2016). Chem. Biol. Technol. Agric..

[9] Tian X., Zhang Z., Yang T., Chen M., Li J., Chen F., Yang J., Li W., Zhang B., Zhang Z. (2016). Front. Microbiol..

[10] Osterman I.A., Komarova E.S., Shiryaev D.I., Korniltsev I.A., Khven I.M., Lukyanov D.A., Tashlitsky V.N., Serebryakova M.V., Efremenkova O.V., Ivanenkov Y.A. (2016). Antimicrob. Agents Chemother..

[11] Volynkina I.A., Zakalyukina Y.V., Alferova V.A., Belik A.R., Yagoda D.K., Nikandrova A.A., Buyuklyan Y.A., Udalov A.V., Golovin E.V., Kryakvin M.A. (2022). Antibiotics..

[12] ISO.

[13] Baranova A.A., Chistov A.A., Tyurin A.P., Prokhorenko I.A., Korshun V.A., Biryukov M.V., Alferova V.A., Zakalyukina Y.V. (2020). Microorganisms..

[14] Gause G.F., Preobrazhenskaya T.P., Terekhova L.P., Maksimova T.S. (1983). Guide for Determination of Actinomycetes: Genera Streptomyces, Streptoverticillium, and Chainia. 1st ed. M.: Nauka, 1983. P. 84–89..

[15] Shirling E.B., Gottlieb D. (1966). Int. J. Syst. Bacteriol..

[16] Zakalyukina Y.V., Osterman I.A., Wolf J., Neumann‑Schaal M., Nouioui I., Biryukov M.V. (2023). Antonie Van Leeuwenhoek..

[17] Williams S.T., Goodfellow M., Alderson G., Wellington E.M.H., Sneath P.H.A., Sackin M.J. (1983). Microbiology..

[18] Zakalyukina Y.V., Birykov M.V., Lukianov D.A., Shiriaev D.I., Komarova E.S., Skvortsov D.A., Kostyukevich Y., Tashlitsky V.N., Polshakov V.I., Nikolaev E. (2019). Biochimie..

[19] Zakalyukina Yu.V., Biryukov M.V., Golichenkov M.V., Netrusov A.I. (2017). Mosc. Univ. Biol. Sci. Bull..

[20] Prjibelski A., Antipov D., Meleshko D., Lapidus A., Korobeynikov A. (2020). Curr. Protoc. Bioinforma..

[21] Brettin T., Davis J.J., Disz T., Edwards R.A., Gerdes S., Olsen G.J., Olson R., Overbeek R., Parrello B., Pusch G.D. (2015). Sci. Rep..

[22] Ondov B.D., Treangen T.J., Melsted P., Mallonee A.B., Bergman N.H., Koren S., Phillippy A.M. (2016). Genome Biol..

[23] Lefort V., Desper R., Gascuel O. (2015). Mol. Biol. Evol..

[24] Zakalyukina Y.V., Pavlov N.A., Lukianov D.A., Marina V.I., Belozerova O.A., Tashlitsky V.N., Guglya E.B., Osterman I.A., Biryukov M.V. (2022). Insects..

[25] Kohlbacher O., Reinert K., Gröpl C., Lange E., Pfeifer N., Schulz-Trieglaff O., Sturm M. (2007). Bioinformatics..

[26] Wang M., Carver J.J., Phelan V.V., Sanchez L.M., Garg N., Peng Y., Nguyen D.D., Watrous J., Kapono C.A., Luzzatto-Knaan T. (2016). Nat. Biotechnol..

[27] van Santen J.A., Poynton E.F., Iskakova D., McMann E., Alsup T.A., Clark T.N., Fergusson C.H., Fewer D.P., Hughes A.H., McCadden C.A. (2022). Nucleic Acids Res..

[28] Van Santen J.A., Jacob G., Singh A.L., Aniebok V., Balunas M.J., Bunsko D., Neto F.C., Castaño-Espriu L., Chang C., Clark T.N. (2019). ACS Cent. Sci..

[29] Segeritz C.-P., Vallier L. (2017). Basic Science Methods for Clinical Researchers.. Elsevier. Academic Press..

[30] Nouioui I., Carro L., García-López M., Meier-Kolthoff J.P., Woyke T., Kyrpides N.C., Pukall R., Klenk H.-P., Goodfellow M., Göker M. (2018). Front. Microbiol..

[31] Goto M., Masegi M.-A., Yamauchi T., Chiba K.-I., Kuboi Y., Harada K., Naruse N. (1998). J. Antibiot. (Tokyo)..

[32] Oja T., Palmu K., Lehmussola H., Leppäranta O., Hännikäinen K., Niemi J., Mäntsälä P., Metsä-Ketelä M. (2008). Chem. Biol..

[33] Naruse N., Goto M., Watanabe Y., Terasawa T., Dobashi K. (1998). J. Antibiot. (Tokyo)..

[34] Ritacco F.V., Eveleigh D.E. (2008). J. Ind. Microbiol. Biotechnol..

[35] Graziani E.I., Ritacco F.V., Bernan V.S., Telliez J.-B. (2005). J. Nat. Prod..

[36] Bycroft B.W., Higton A.A., Roberts A.D. (1988). Dictionary of antibiotics and related substances. // London ; New York. 1988. P.944..

[37] Van Pée K.-H., Lingens F. (1984). FEBS Lett..

[38] Söhngen C., Podstawka A., Bunk B., Gleim D., Vetcininova A., Reimer L.C., Ebeling C., Pendarovski C., Overmann J. (2016). Nucleic Acids Res..

[39] Schwyn B., Neilands J.B. (1987). Anal. Biochem..

[40] Poumale H.M.P., Ngadjui B.T., Helmke E., Laatscha H. (2006). Z. Für Naturforschung B..

[41] Song Z.-M., Zhang J.-L., Zhou K., Yue L.-M., Zhang Y., Wang C.-Y., Wang K.-L., Xu Y. (2021). Front. Microbiol..

[42] Sagurna L., Heinrich S., Kaufmann L.-S., Rückert-Reed C., Busche T., Wolf A., Eickhoff J., Klebl B., Kalinowski J., Bandow J.E. (2023). Antibiot. Basel Switz..

[43] Tian W., Wang C., Li D., Hou H. (2020). Future Med. Chem..

[44] Pavlova J.A., Khairullina Z.Z., Tereshchenkov A.G., Nazarov P.A., Lukianov D.A., Volynkina I.A., Skvortsov D.A., Makarov G.I., Abad E., Murayama S.Y. (2021). Antibiotics..

[45] Liu Y., Zhong Y., Tian W., Lan F., Kang J., Pang H., Hou H., Li D. (2019). Anticancer. Drugs..

[46] Hitosugi T., Zhou L., Elf S., Fan J., Kang H.-B., Seo J.H., Shan C., Dai Q., Zhang L., Xie J. (2012). Cancer Cell..

[47] Wang D., Wang S., Liu Q., Wang M., Wang C., Yang H. (2013). Oncol. Rep..

[48] Stanojković T., Marković V., Matić I.Z., Mladenović M.P., Petrović N., Krivokuća A., Petković M., Joksović M.D. (2018). Bioorg. Med. Chem. Lett..

[49] Ijaz T., Tran P., Ruparelia K.C., Teesdale-Spittle P.H., Orr S., Patterson L.H. (2001). Bioorg. Med. Chem. Lett..

